# Natural Disasters and Injuries: What Does a Surgeon Need to Know?

**DOI:** 10.1007/s40719-018-0125-3

**Published:** 2018-03-23

**Authors:** Sofia Bartholdson, Johan von Schreeb

**Affiliations:** Centre for Research on Health Care in Disasters, Health System and Policy Research, Department of Public Health Sciences, Karolinska Institute, Stockholm, Sweden

**Keywords:** Natural disasters, Trauma surgery, Wounds and injuries, Emergency medicine, Disaster medicine, Disaster victims

## Abstract

**Purpose of review:**

Natural disasters have injured more than 2 million people in the last 10 years and led to significant international medical relief deployment. Knowledge of expected injury patterns following these disasters is an important part of planning for type and size of outside surgical assistance. This review aims to summarize what is known about injury patterns following natural sudden-onset disasters (SODs).

**Recent findings:**

Several systematic reviews have concluded that data on injury patterns and surgical needs following natural SODs is scarce. Studies on earthquakes indicate that earthquakes generate large numbers of injured, out of which limb injuries are most common. Tsunamis, floods, storms, and wildfires do not generate a significant burden of injuries in relation to numbers affected.

**Summary:**

Earthquake may require surgical assistance, especially for limb injuries; therefore, mainly orthopedic and plastic surgeries are priority specialist areas. Major injuries seem to be few in other natural disasters. However, more detailed data is needed on specific injury patterns to determine if additional surgical assistance is needed and to what extent it is needed to cater for normal surgical conditions if existing health care has seized to function.

## Introduction

In the last 10 years, natural disasters have killed 760,000 people, injured 2 million and affected more than 2 billion people [1]. Natural disaster caused mortality and morbidity is likely to increase in coming years due to climate change [2, 3].

**Fig. 1:**
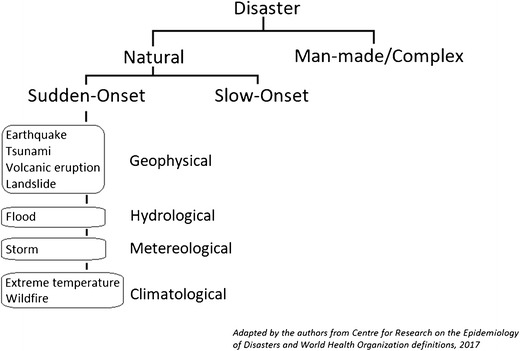
Definition of sudden onset natural disasters, adopted from CRED and WHO and adapted by the authors

A disaster is defined by the Centre of Research on the Epidemiology of Disasters (CRED) as “a situation or event which overwhelms local capacity, necessitating a request to a national or international level for external assistance; an unforeseen and often sudden event that causes great damage, destruction and human suffering.” [4] Natural disasters, as compared to man-made disasters, are caused by natural phenomena. Several natural disaster classifications exist. CRED uses a cause-based classification where natural disasters are divided into six main groups: biological, geophysical, hydrological, meteorological, climatological, and extra-terrestrial [4]. Natural disasters can also be divided into slow or sudden-onset. WHO defines sudden-onset disasters (SODs) as disasters “for which there is little or no warning” (Fig. 1) [5].

Recent natural SODs in low- and middle-income countries have led to significant international medical relief deployment [6–8]. However, this response has been critiqued for being of low quality and not adapted to existing health needs [9]. In order to address this, a classification system and standards for emergency medical teams (EMT) was defined by WHO in 2013 [10]. EMTs deploying to international disasters need to adhere to these standards and be WHO-verified beforehand [11]. To ensure that EMTs are equipped and staffed to cater for the health needs, knowledge about the health effects of different disasters is essential in addition to context-specific information. From a surgical perspective, knowledge of injury patterns in different disasters can help both local health care coordination, as well as defining whether international EMTs are needed and adapt their deployment. This study aims to summarize current knowledge about surgical injury patterns following different types of natural SODs.

## Methods

This literature review was done using online database searches with the search words “injury,” “sudden onset disaster,” “surgery,” “trauma,” and specific natural disaster terms in various combinations. In addition, articles from reference lists from retrieved articles were identified. Relevant articles were read, and data on injury was extracted and compiled using an adapted CRED categorization.

### Injuries Following Natural SODs

Overall injury patterns are poorly studied in natural SODs. Population-based epidemiological studies on frequency and type of injuries are few. Most studies and reports are based on health facility collected data which creates significant bias by leaving out a great number of the affected population. In addition, level of exposure to the harm of the sudden onset event varies and is not specified. This means that the available data only gives a faint picture of the real burden of injuries. However, we have attempted to present and discuss the best available evidence that may provide basic guidance to define if and to what extent there is need for specific surgical injury management following different SODs.

### Wildfires

Studies on adverse health events among firefighters during wildfires show that burn injuries are uncommon and that this group is more prone to other health problems such as mild extremity injuries, eye irritation, and smoke inhalation [12, 13]. They probably have an increased risk of mortality, mostly because of inhalation injuries [14, 15].

Few studies have studied injury types and patterns caused by vegetation or wildfires in the general population. One study assessed hospital visits related to a big 1991 urban wildfire in California. A total of 4% presented with burns, 1% major trauma, 7% minor trauma, and 4% simple fractures with no further description. In contrast, bronchospastic and irritative smoke reactions were significantly more common, constituting 31 and 20% of the total patient visits, respectively [12].

### Landslides

Landslides are one of the deadliest natural disasters, with a death-injury ratio of 4,5:1 [1] Little is known about specific injury patterns following landslides as few studies have been conducted [16•]. Probably the most common cause of death during landslides is suffocation, with some additional deaths are caused by injuries from debris and rockfalls [16•, 17].

In 2002, Chuuk in Micronesia suffered a landslide which killed 43 people. Injuries in survivors seemed to be minor and mostly included contusions and lacerations [18]. One study of a landslide in Japan 2013 described 49 patients, of which 34 were triaged as black, and the remaining 15 were hospital-treated. Seven of these had mild injuries such as bruises or dermabrasion. Of the higher triaged patients, seven suffered lung injuries including pneumothorax and pulmonary contusion. Pelvic ring fractures, thoracic fractures, and facial fractures coexisted [19]. A report from a landslide in 2011, Brazil, states that all deaths were due to suffocation (mud burial or drowning) and that the most common injury in survivors was mild lower extremity injury such as wounds or lacerations. Only 11 of the 138 admitted patients needed surgery [17].

### Volcanic Eruptions

There are few studies on mortality causes and morbidity from volcanic eruptions. Life-threatening health effects include ash inhalations with suffocation, scalding, and burns from pyroclastic density currents (PDCs) and inhalation of lethal toxic gas [20].

PDCs can spread fast over large areas and hold temperatures of 200 to 500 °C [21]. These cause lethal burn injuries and are the most common cause of death in volcanic eruptions. The three following most common causes of death are from indirect causes (starvation and disease), tsunamis and lahars [22]. A volcanic eruption in Colombia in 1985 generated lahars which claimed 25,000 lives [23].

One recently published study studied burn injuries following two volcanic eruptions in 1994 and 2010 on Java. Out of 106 admitted patients, 44 and 16% of the patients, respectively, sustained burn injuries with a total body surface area of more than 80%. Of all the burn-injured patients, only 29% in 1994 and 37% in 2010 survived for discharge [24]. A study done during a 1989 ashfall in the USA showed increases in asthma and acute bronchitis. Of 25 people found dead, the cause of death was suffocation in 76%, and thermal injury or head trauma in 12%, respectively [25].

### Extreme Temperatures

Cold waves, or cold spells, have been shown in a 2015 systematic review and meta-analysis by Ryti et al. to increase overall mortality [26]. Very little is known about potential injuries during cold waves. A few studies on ice storms in Canada and the USA exist, but the temperatures during these events are not necessarily lower than normal. Ice storms seem to cause falls leading to contusions and fractures [27]. However, studies have not been able to show an absolute increase in these injuries compared to similar winter periods [28, 29].

Heatwaves increase the amount of emergency department visits and have been shown to increase overall mortality, especially in the elderly population [30–32]. A 10-day heatwave in Greece 1987 resulted in 2960 heat-related admissions with a 31% mortality rate [31]. Cardiac diseases including stroke, respiratory illness, renal failure, and sepsis have all been found to become more frequent during hot periods [32, 33]. In terms of excess trauma and injuries, there are no available data.

### Earthquakes

Earthquakes are the main killer of all natural disasters on an annual basis. In the last 10 years, 350,000 people have died, and more than 1,000,000 people have been injured [1]. Earthquakes might also generate tsunamis, which can increase the death toll [34].

Earthquakes seem to have an approximate average death-injured ratio of 1:3–4, but it varies [1]. One reason may be the lack of a common definition of what is an injury. High-magnitude earthquakes might cause more crush injuries and burying than low magnitude [35•]. Building damage is a major cause of death and injury in earthquakes, especially where solid building materials such as concrete are used, and buildings are not adapted to withstand ground motion [36]. In addition, the time of onset influence the death toll. Earthquakes cause infrastructural damage, damage to health facilities, and disruption of transportation. This might cause traffic injuries and death, but also restrict rescue efforts and increase the vulnerability of the affected population [36, 37]. Among the instant deaths, severe trauma with head injury, asphyxiation, and/or shock is reportedly the most common cause of mortality [38].

In a review from 2017, MacKenzie et al. studied types and locations of orthopedic injuries following earthquakes. They found that fractures accounted for 65% of the total injuries and that 59% of all injuries affected the lower extremities. Other orthopedic injuries included crush injuries, compartment syndromes, major soft-tissue injuries, and crush syndromes [39•].

A recent systematic review by Bortolin et al. analyzed 34 articles and concluded that lower extremities were the most common fracture location (42%). Spinal column fractures accounted for 17% of the total injuries, and more than 4% of these were spinal injuries, while 10% of fractures involved the pelvic ring. They also found that fracture incidence seemed to correlate with the Richter Magnitude Scale, with a high-energy earthquake causing more fractures [35•].

A review from 2013 by Missair et al. found a global incidence of limb injuries in survivors of 54%, with lower extremity injuries being most common. Cranial, thoracic, and abdominal injuries accounted for less than 30% of the total injuries combined [40]. These injuries might not be reported as many of them lead to death prior to arrival at hospital.

Burn injuries are relatively rare, but more common in rural areas of low- and middle-income countries, where basic cooking methods and heating is used. Earthquakes occurring during common cooking times might also increase the burn incidence [35•, 41].

It is not clear if and how pediatric injury patterns differ from those in adults, but it seems as extremity fractures is most common also in this group [42]. One study from Haiti found that children had a higher surgery rate than adults and a higher percentage of femoral fractures [43].

### Tsunamis

Tsunamis are most often induced by earthquakes, but volcanic eruptions and other phenomena affecting large volumes of water might also create tsunamis [34, 44]. In unprotected areas, they can cause significant damage. This was the case in the 2004 tsunami which killed more than 220,000 people and affected 2.4 million [1].

The death-injured ratio in tsunamis is approximately 4:1—the opposite compared to earthquakes [1].

Randomly selected, displaced households in the Aceh province were surveyed by Doocy et al., after the 2004 tsunami. The weighted total injury rate was 8.8%. Of these, 75% were lacerations, of which more than two-thirds were infected. Even though the authors state that the injury severity might be overestimated in this study, 92% of all injured fully recovered [45].

Several tsunami disasters show that survivors are relatively mildly injured. This is thought to be explained mainly by that the risk of drowning is significant for severely injured. Following tsunamis, the number of patients to treat and the severity of their injuries have been overestimated [44, 46, 47]. Mild extremity trauma including lacerations, and also minor fractures, has been reported [44, 45, 48•]. Due to delayed care, tropical climate and seawater-contaminated wounds infections are common and typically have a broad bacterial spectrum [44, 49]. The most common adverse health effect after a tsunami is pneumonitis due to asphyxiation of seawater [47, 49].

### Floods

Floods are the most common natural disasters worldwide [1]. Drowning is the most common cause of death following floods [44, 50, 51].

A systematic review by Saulnier et al. 2017 found that very few studies have studied injuries caused specifically by floods [52•]. One study from the 2008 flood in Vietnam interviewed 871 households about adverse health effects during the first post-disaster month. Only 27 injuries were reported, out of which the majority were minor cuts and lacerations caused by falls [53]. Acute physical injuries tend to be few and mild, including sprains, lacerations, and in some cases fractures [44, 51, 53]. Most of the injuries are related to clean-up, rather than from flood impact. Communicable disease and wound infections are common because of contaminated water and malfunctioning air-conditioning and heating [44]. Long-term health issues such as spread of communicable diseases and compromised access to health care facilities cause more problems than acute injuries [44, 53].

### Storms

Injury patterns during storms are not much studied and lack data uniformity. It is suggested that most injuries and deaths during cyclonic storms are related to subsequent floods, most often created by heavy rains [54]. As mentioned in the above section, flood injuries seem to be predominantly made up by drownings and near-drownings, with a low number of extremity injuries [50].

One study assessed emergency visits following two 2008 hurricanes in the USA. Out of the 3863 patient visits, less than 10% were labeled as injuries (including sprains, cuts, falls, and contusions). Of these, lacerations and cuts represented more than 60%, while more serious injuries such as fractures and concussions were only 1% each [55]. In Hong Kong, tropical cyclone-related illness has since 2003 regularly been entered into a database. A study from 2011 analyzed part of the database and found 460 cyclone-related injuries from 15 emergency departments between 2004 and 2009. They concluded that the most common injuries were contusions/abrasions and lacerations and the most common locations the head and extremities. Most injuries were minor, with only 2.8% triaged as high category [56].

A systematic review performed in 2013 by Doocy et al. showed that direct deaths accounted for 56% of the total deaths, the rest were due to indirect causes. The most common causes of direct death were drowning (59%) and trauma (39%). Indirect death was mostly due to trauma including vehicle accidents, burns, and electrocution. Fifteen included studies provided injury pattern data. The most common injuries included minor lacerations and wounds, contusions and blunt trauma, motor vehicle accidents, and animal bites/stings. Total injury rates in the affected populations were found to be between 3.8 and 4.5% in three included, population-based studies [57].

## Discussion

Our study found that the injury pattern varies greatly between different natural SODs. However, there is a lack of population-based epidemiological studies on physical injuries and surgical needs.

Earthquakes are the only SOD that cause a significant burden of injuries, mainly of the limb. Because of large numbers of extremity injuries, including wound and fractures, there is a demand for orthopedic and plastic surgery. The main challenge is to ensure rapid surgical care for critical injuries. Outside assistance arrives late, and transportation to functional hospitals is too long to save lives of the most critically injured. This may explain why relatively few abdominal and head/neck injuries are reported during earthquakes. General surgeons are needed for wound management and debridement but also to cater for all non-disaster-related surgeries, including cesarean sections. Physiotherapists and orthopedic technicians are essential for early rehabilitation [58, 59].

The need for trauma surgery following tsunamis, storms, floods, and wildfires is low. This is probably also true for most landslide and volcanic eruption disasters, even though a few studies suggest some severe trauma and burn injuries. Injuries following these disasters are generally few compared to the total numbers affected, and mostly consist of lacerations and minor contusions. It may be assumed that these lacerations or wounds easily get infected, and it is reasonable to believe that surgical procedures foremost will include basic debridement and leaving the wound open for delayed primary suture. Other health care needs such as care for communicable diseases remain more important. Experience show that the most important resources after these disasters are not surgeons, but nurses with wound care skills and general practice doctors with basic surgical skills and ability to treat communicable and non-communicable diseases.

There is no data on injuries during heatwaves and very limited evidence regarding injury patterns during cold waves. Extreme temperatures probably do not cause any significant surgical burden, but more studies are needed.

Secondary injuries are quite common after disasters during clean-up, including falls and traffic accidents, but generally minor. Infrastructural and property damage causing a general lack of health care facilities and medicine is a threat of health in the affected population. When existing health facilities have been destroyed, outside surgical assistance may be needed to cater both for the normal burden of disease and the few severely injured.

## Conclusion

There is limited population-based data on type of injuries following natural SODs. Existing studies indicate that besides earthquakes, major injuries are not a significant problem, while bruises and lacerations may render basic wound management. To determine whether outside surgical assistance is needed, information on expected injury pattern, estimated number of injured, and existing health care resources are necessary.
